# Observations on the Diseases of the Pancreas

**Published:** 1814-02

**Authors:** Thomas Sewall


*For the Medical and Physical Journal.*

### *Observations on the Diseases of the Pancreas; by* THOMAS SEWALL, M.D.

THE diseases of the pancreas are few. Scirrhus, and inflammation, with its terminations in abscess and ulceration, are nearly the only diseases with which it has been found affected. The former is much the most common, and more particularly the object of this essay.

The pancreas, like all other glandular parts, when it assumes the scirrhous state, loses entirely its natural structure, and is converted into a hard uniform white or brown mass, intersected by membranous septa. It is, however, not very uncommon to meet with this organ considerably enlarged and approaching to a state of induration, without any sensible derangement in its organization. This seems, however, only the commencement of a process by which it loses entirely its natural structure, and becomes truly scirrhous.

The following cases contain an account of the symptoms which accompany an enlarged and scirrhous affection of this organ with the appearances on examination after death. The two first are taken in substance from Morgagni's third book on diseases of the belly.

The first is that of a noble matron of Padua, who from her very birth was troubled with an almost constant vomiting, which which returned regularly every morning, and two hours after dinner. When she made any exertion to suppress the vomiting at its usual time of accession, great uneasiness was produced in the stomach, which continued till the offending matter was dislodged. The bowels were generally costive, and could only be kept free by the use of purgative medicine. This with moderate bleeding were the only remedies employed in the course of her life.

On dissection, the body was emaciated, and without any œdematous tumor. The abdomen contained a considerable quantity of water. The omentum was destitute of fat, and the stomach and intestines much contracted. The pancreas was the only one of the abdominal viscera which exhibited any marks of disease. This, though of its ordinary size, was hard and white, with a complete change in its structure, consisting of a large number of distinct lobules quite destitute of moisture.

The second case is that of a man, otherwise robust and healthy, who, without any evident preceding cause, was troubled with a continual endeavor to vomit, by which every thing was discharged from the stomach soon after it was taken down. He was also affected with a constant and severe pain in the region of the stomach.

On opening the abdomen after death, all the viscera appeared in a healthy state, except the pancreas; this was considerably enlarged, and almost of a cartilaginous hardness. Its surface was universally irregular, from tubercles of a considerable size, over its whole extent. These, I believe, are the only cases of an original affection of the pancreas mentioned by Morgagni, and but little is to be met with in any other author, immediately relative to this subject.

The two following cases, having recently occurred under my own observation, are related particularly with a view to their symptoms and appearances on dissection.

Mr. S. S., a young man of slender constitution, of studious and sedentary habits, a few years before his death was seized with an obtuse pain, deep seated and fixed in the region of the epigastrium; with an incurvation of the body forward, and an inability to sit or stand erect without greatly increasing its severity. In a few months these symptoms were accompanied with dyspepsia, great acidity of the stomach, and an occasional vomiting. His appetite was generally good, but nothing could be retained long enough by the stomach to undergo digestion except milk, which constituted the greatest part of his diet ever after the attack, till his decease.

The remedies resorted to in the course of the disease were emetics; emetics, cathartics, and blistering over the region of the stomach. The latter was generally accompanied with greater relief than was experienced from the application of any other remedy, but this was only temporary. These symptoms, (pain, acidity of the stomach, and vomiting,) continued more or less severe till a few months before his death, when a pulmonary consumption came on, that terminated fatally.

The day after his death the thoracic and abdominal viscera underwent a careful inspection. The general appearance of the body was that of great emaciation without any œdematous affection.

On opening the thorax it was found to contain a small quantity of water, the lungs adhering to the pleura, for nearly their whole extent of surface, hard and unyielding, and in many places ulcerated and affected with tubercles. On laying open the abdomen, it was found to contain about three quarts of water. The omentum was destitute of fat, and the stomach and intestines greatly contracted. The liver, spleen, and urinary organs, all exhibited a healthy appearance. The pancreas was the only one of the abdominal viscera in which there was any mark of disease. This was enlarged to about twice its natural size, and scirrhous, particularly its right extremity, which embraced the duodenum, and pressed so firmly on the pylorus, that its orifice would scarcely admit of the introduction of a common-sized catheter.

The second case which came under my notice, was that of Mr. B. S., a young man, aged twenty-seven, who had been confined most of his life to a sedentary employment, which gave him but little opportunity for the enjoyment of active exercise or good air. About one year before his death he was affected with a tumefaction of the parotid and submaxillary glands, also of some of the lymphatic glands of the neck. This suddenly subsided, and soon after a considerable tumor made its appearance in the epigastrium, accompanied with severe deep-seated pain and an almost constant vomiting. There was, as in the preceding case, an incurved position of the body, and inability to sit, or stand erect, without greatly increasing the severity of the pain. Nothing could be retained by the stomach but liquid food, and that only when taken in small quantities and at short intervals. The bowels were usually costive, aud could only be kept free by the use of cathartics and enemas. These, with blistering over the stomach, opium, and lime-water, were the most effectual remedies in alleviating these distressing symptoms. But their effect was only palliative; great emaciation and debility ensued, which finally terminated in death.

On

On dissection, the thoracic viscera were carefully examined, and exhibited not the smallest vestige of disease.

On laying open the abdomen it was found to contain a small quantity of water. The omentum was destitute of fat, and the stomach and intestines contracted to half their original size. In the liver, spleen, and kidneys, there was no remarkable deviation from their ordinary healthy appearance. On examining the pancreas, it was found enlarged, and nearly three times its natural size, with its left extremity extending into the left lumbar region, and its right, pressing firmly on the duodenum and small extremity of the stomach, and thereby nearly obliterating its pyloric orifice. Its surface was universally irregular, hard, and unyielding, and in many places exhibited in its appearance the scirrhous structure.

From the foregoing cases, pain in the epigastrium, vomiting, and acidity of the stomach, appear to constitute the most remarkable indications of an enlarged and scirrhous affection of the pancreas.

The pain is generally one of the earliest and most distressing symptoms of the disease. It is usually of a heavy obtuse kind, deep seated, and without any considerable change in its situation. This was remarkably true in the above cases; it is also spoken of by Riverius and some other authors, as a principal symptom of the disease.*

Vomiting is another symptom which strongly indicates an affection of the pancreas. This was a leading feature of the disease in each of the above cases; it is also mentioned by Morgagni and Bonetus, as constituting the principal indication of an affection of the pancreas in all those cases, which they examined after death.

Great acidity of the stomach is generally present, but should be regarded only as a secondary symptom, arising from some derangement in the functions of digestion.

Whether this peculiar state of the stomach arises simply from the mechanical effect of the pancreas on it, from its connection by sympathy, or from a deficiency or some change

---

* Pain. To what cause this should be attributed is not certain. Probably to increased vascular action. It is a fact generally admitted, that the blood-vessels of an inflamed part become, by the force of their own action, increased in their capacity and distended with blood. Take in connection with this, the circumstance that the blood-vessels of a scirrhous organ are diminished in their capacity, and incapable of easy distension, from the resistance made by the scirrhus, through which they ramify, we cannot be at a loss for an explanation.

in the pancreatic secretion, is an interesting question difficult to decide; perhaps neither should be wholly neglected. On mechanical principles we can readily account for the vomiting and acidity, from the pressure of an enlarged and scirrhous pancreas on the stomach and pylorus, retarding the passage of the alimentary mass into the intestines. At the same time, the sympathy excited between these two organs, no doubt renders the stomach more irritable and easily excited into action by its contents.

With respect to the functions of the pancreas, in its diseased state, we have but little knowledge. We have no evidence that a change in the structure of this organ is accompanied by any specific change in the nature of its secretion. On the other hand, in the cases in which Morgagni speaks of the dark vomiting, in this disease, the pancreas, when cut into, appeared dry, and afforded but little of its natural secretion. The vomiting, therefore, and the peculiar character of the matter thrown up, appears to proceed rather from a deficiency of pancreatic juice, than from any specific change in its quality.

The incurvated state of the body appears also worthy of notice. This is probably chosen from its relaxing the abdominal muscles, enlarging the cavity of the abdomen, and thereby taking off from the diseased pancreas the pressure of the neighbouring viscera.

The above symptoms, so far as experience teaches, constitute the principal indications of an enlarged and scirrhous affection of the pancreas;* but unfortunately they are not invariably referable to an affection of this organ. Certain affections of the stomach, small intestines, liver, and spleen, sometimes embrace them. It is, however, generally not difficult when we take into view all the circumstances accompanying an original affection of these organs, to distinguish between them with a tolerable degree of certainty.

Inflammation, with its terminations in abscess and ulceration, are said sometimes to affect the pancreas; but we are not in possession of a set of symptoms, which, in the present state of our knowledge, enable us to discriminate between affections of this kind, and its scirrhous state.

Some few instances have occurred, in which worms and calculus have been taken from the pancreatic duct; † but

---

* I am informed by one of our first American practitioners, (Professor Warren,) that a tumor in the epigastrium, with a strong pulsation, is generally an attendant on scirrhous pancreas.

Perhaps this is the only invariable pathognomonic symptom of the disease.

these these are extremely rare, and unaccompanied with any symptoms which enable us to foretell their existence.

The treatment of scirrhous affection of the pancreas, it is evident, can be only palliative. The principal indications are to alleviate the pain, restrain the vomiting, and correct the acidity of the stomach.—*New England Journal of Medicine and Surgery, Jan.* 1813.

